# Supplementary material for: Differences in CD80 and CD86 transendocytosis reveal CD86 as a key target for CTLA-4 immune regulation
Source: Nat Immunol. 2022 Aug 23;23(9):1365–78. doi: 10.1038/s41590-022-01289-w (PMC9477731; doi:10.1038/s41590-022-01289-w)
Supplement: Supplementary file 1 — Supplementary Fig. 1. [file 41590_2022_1289_MOESM1_ESM.pdf]

nature portfolio

https://doi.org/10.1038/s41590-022-01289-w

Supplementary information

Differences in CD80 and CD86 transendocytosis reveal CD86 as a key target for CTLA-4 immune regulation

In the format provided by the authors and unedited

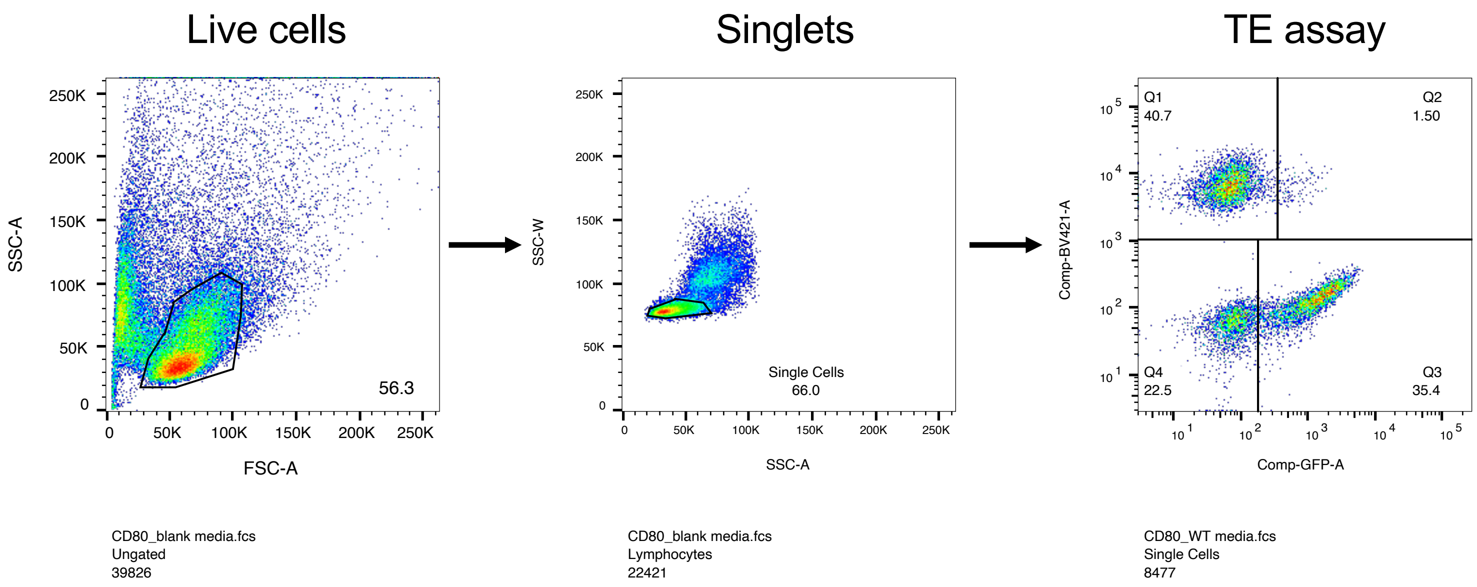
Live cells
Singlets
TE assay
250K
200K
150K
100K
50K
0
SSC-A
FSC-A
50K
100K
150K
200K
56.3
SSC-W
Single Cells
66.0
Comp-BV421-A
Comp-GFP-A
Q1
40.7
Q2
1.50
Q4
22.5
Q3
35.4
CD80_blank media.fcs
Ungated
39826
CD80_blank media.fcs
Lymphocytes
22421
CD80_WT media.fcs
Single Cells
8477
