# Supplementary material for: Differences in CD80 and CD86 transendocytosis reveal CD86 as a key target for CTLA-4 immune regulation
Source: Nat Immunol. 2022 Aug 23;23(9):1365–78. doi: 10.1038/s41590-022-01289-w (PMC9477731; doi:10.1038/s41590-022-01289-w)
Supplement: Source Data Fig. 2 — Unprocessed immunoblots. [file 41590_2022_1289_MOESM6_ESM.pdf]

Figure 2a (cropped for CTLA4 WT)

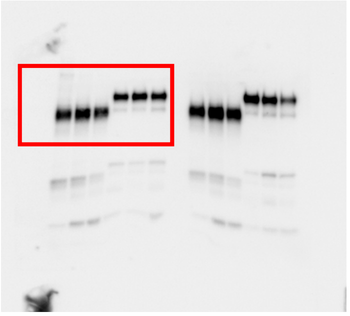

WCL: GFP

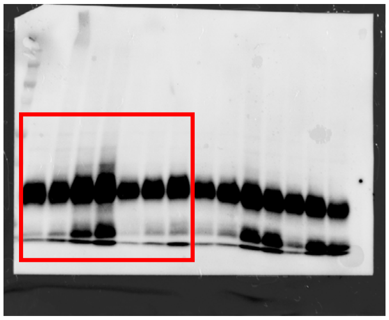

WCL: CTLA4

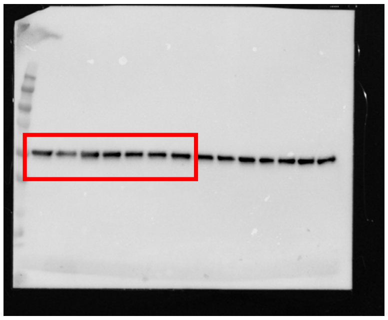

WCL: Tubulin

Figure 2c

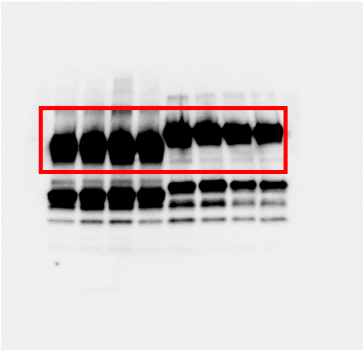

IP: GFP
WB:GFP

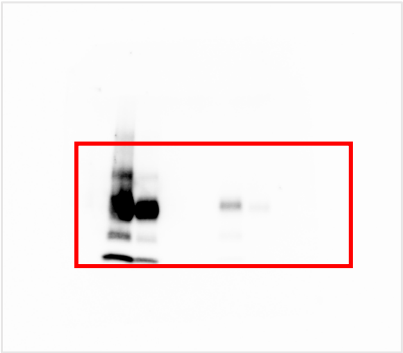

IP: GFP
WB:CTLA4

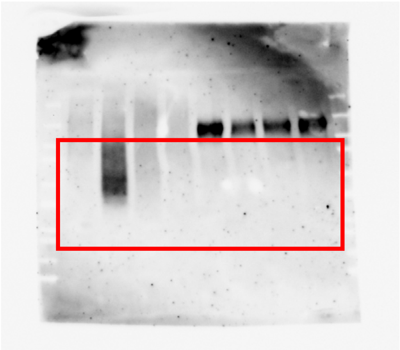

IP: GFP
WB:Ubiquitin

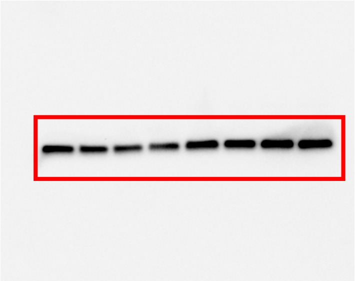

WCL: Tubulin

Figure 2d

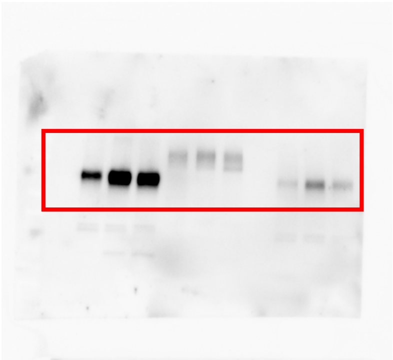

IP: Ubiquitin
WB:GFP

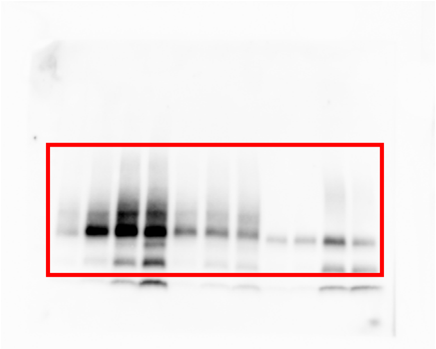

IP: Ubiquitin
WB:CTLA4

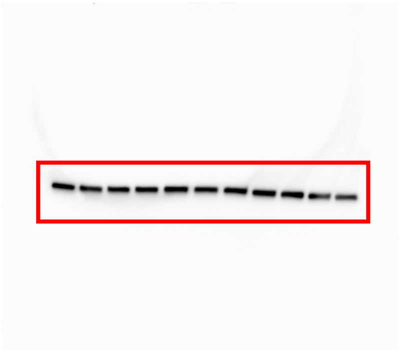

WCL: Tubulin

Figure 2e

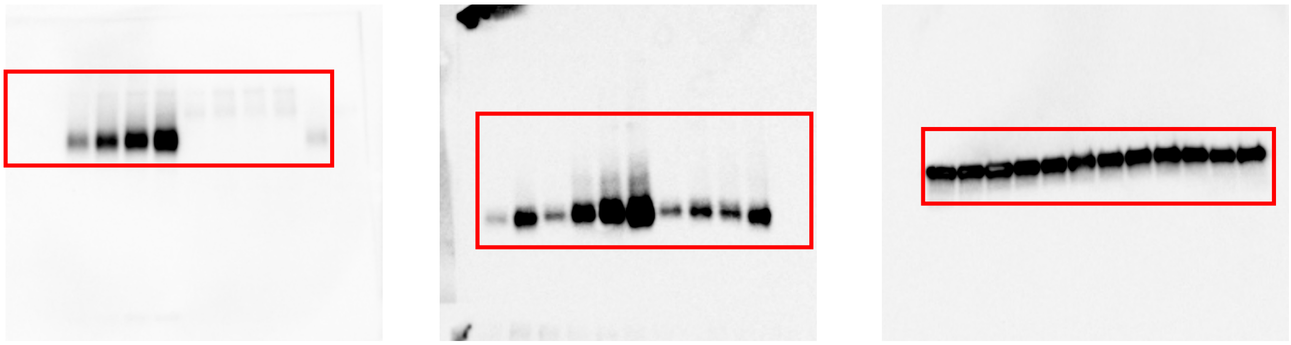

IP: Ubiquitin
WB:GFP

IP: Ubiquitin
WB:CTLA4

WCL: Tubulin

Figure 2f

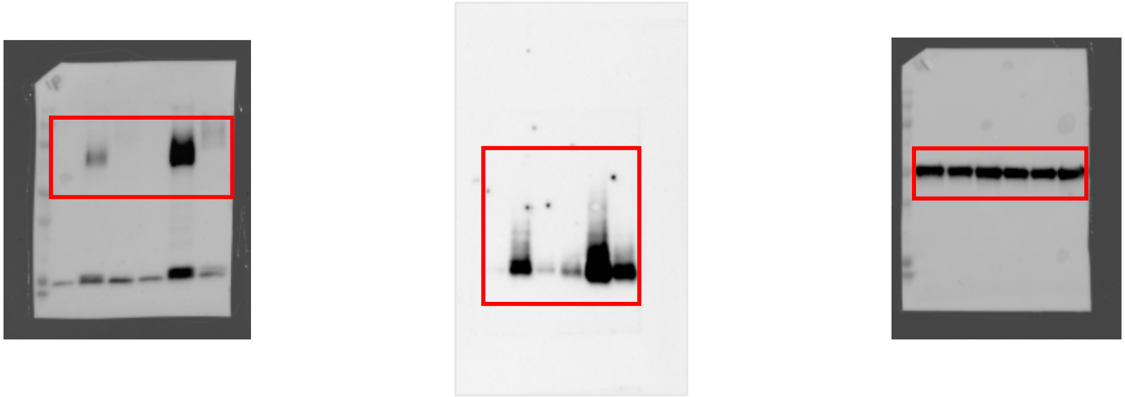

IP: Ubiquitin
WB:GFP

IP: Ubiquitin
WB:CTLA4

WCL: Tubulin
