# Supplementary figures and images for: Differences in CD80 and CD86 transendocytosis reveal CD86 as a key target for CTLA-4 immune regulation
Source: Nat Immunol. 2022 Aug 23;23(9):1365–78. doi: 10.1038/s41590-022-01289-w (PMC9477731; doi:10.1038/s41590-022-01289-w)

Figure 3c

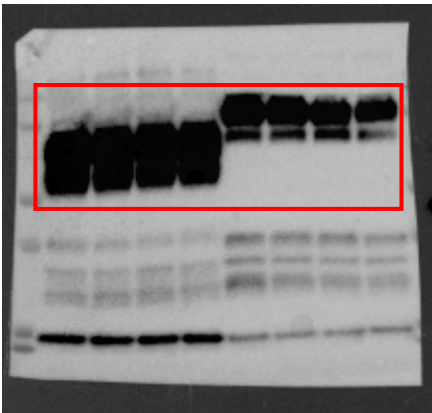

WCL: GFP

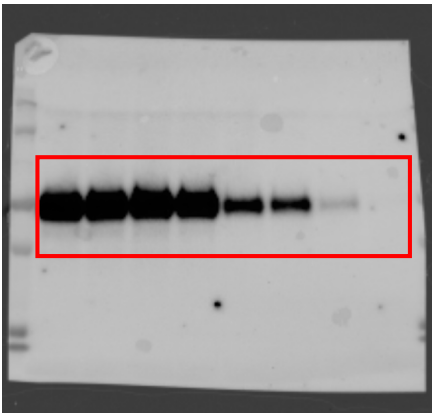

WCL: CTLA4

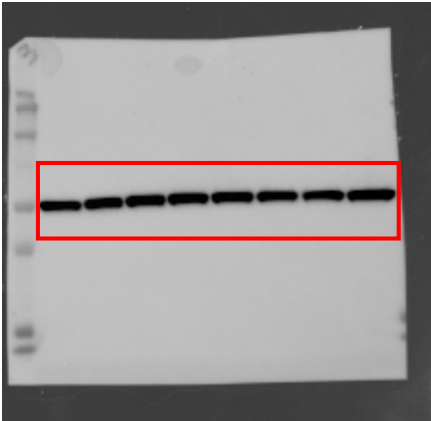

WCL: Tubulin

Supplement: Source Data Fig. 3 — Unprocessed immunoblots. [file 41590_2022_1289_MOESM8_ESM.pdf]

Extended Data 2a

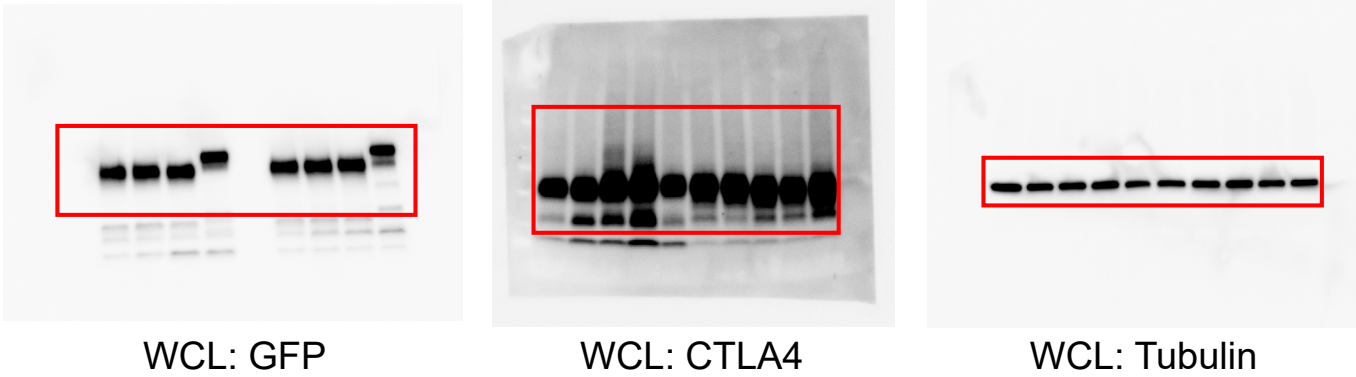

Extended Data 2b (cropped for CTLA4 Kless)

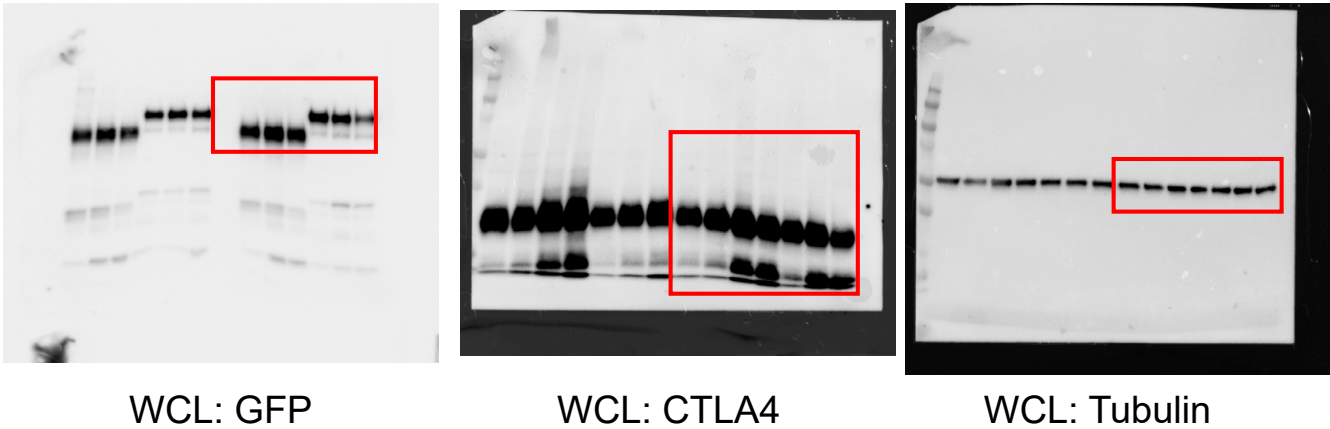

Extended Data 2c

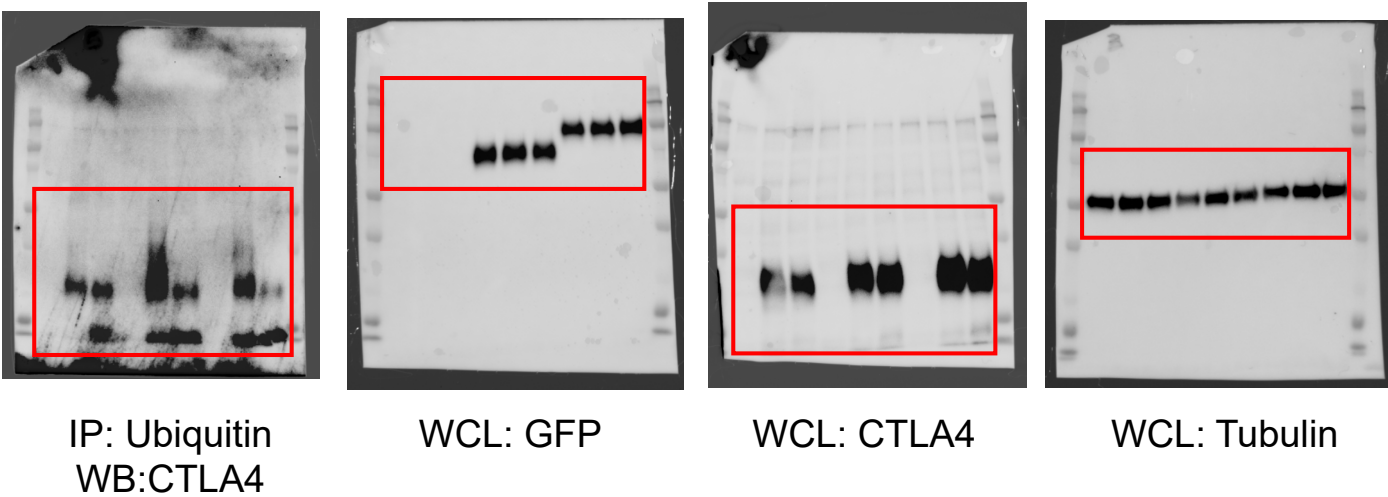

Supplement: Source Data Extended Data Fig. 2 — Unprocessed immunoblots. [file 41590_2022_1289_MOESM13_ESM.pdf]
