# Supplementary material for: Differences in CD80 and CD86 transendocytosis reveal CD86 as a key target for CTLA-4 immune regulation
Source: Nat Immunol. 2022 Aug 23;23(9):1365–78. doi: 10.1038/s41590-022-01289-w (PMC9477731; doi:10.1038/s41590-022-01289-w)
Supplement: Source Data Extended Data Fig. 6 — Unprocessed immunoblots. [file 41590_2022_1289_MOESM16_ESM.pdf]

Extended Data 6c

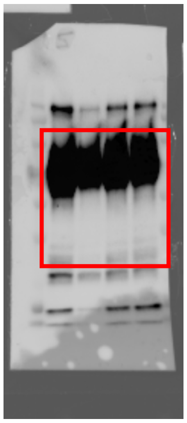

IP: GFP
WB:GFP

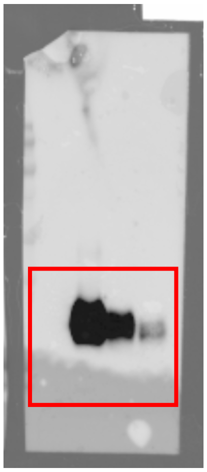

IP: GFP
WB:CTLA4

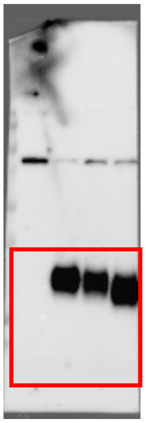

WCL: CTLA4

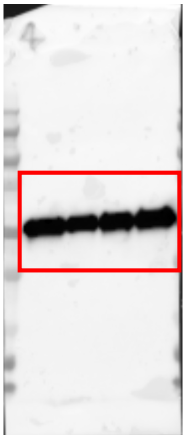

WCL: Tubulin

Extended Data 6d

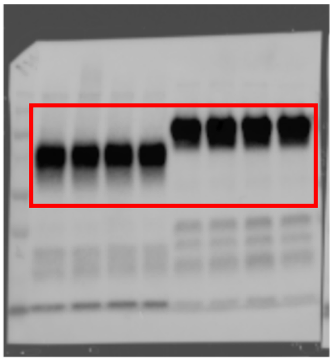

Left: WCL: GFP

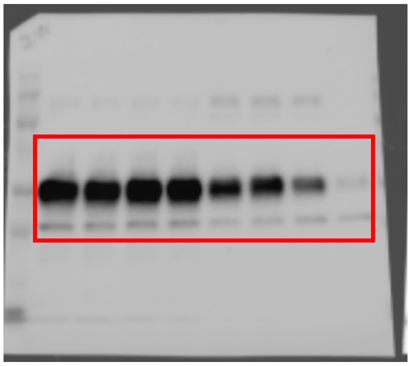

Left: WCL: CTLA4

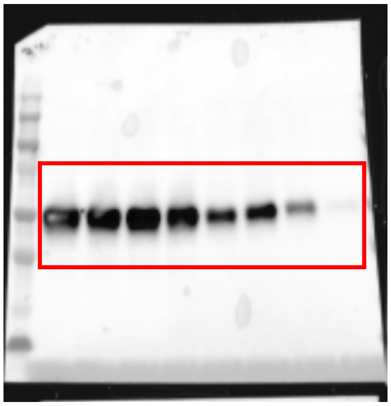

Left: WCL: IgG-CTLA4

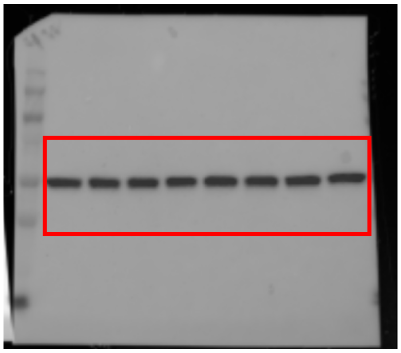

Left: WCL: Tubulin

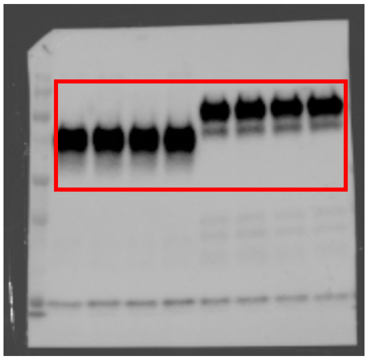

Right: WCL: GFP

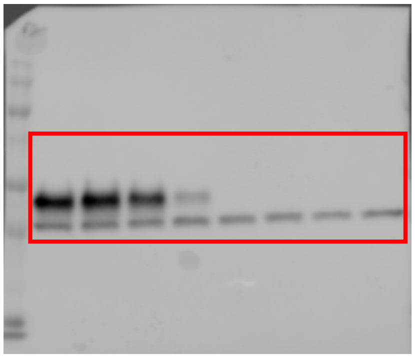

Right: WCL: CTLA4

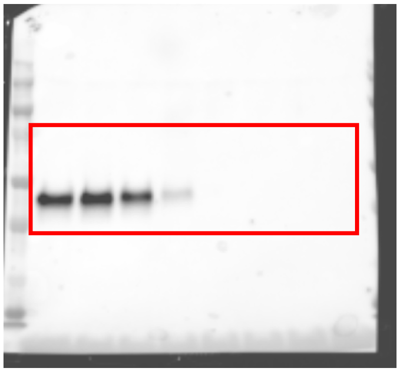

Right: WCL: IgG-CTLA4

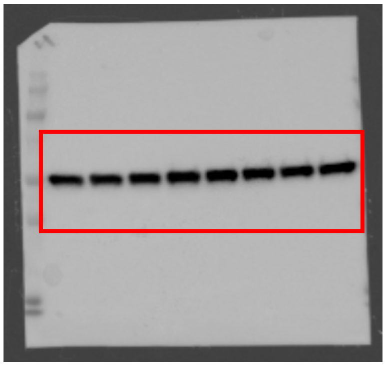

Right: WCL: Tubulin
